# Following Mitochondrial Footprints through a Long Mucosal Path to Lung Cancer

**DOI:** 10.1371/journal.pone.0006533

**Published:** 2009-08-06

**Authors:** Santanu Dasgupta, Rex C. Yung, William H. Westra, David A. Rini, Johann Brandes, David Sidransky

**Affiliations:** 1 Department of Otolaryngology-Head and Neck Surgery, Johns Hopkins University, Baltimore, Maryland, United States of America; 2 Division of Pulmonary & Critical Care Medicine, Johns Hopkins University School of Medicine, Baltimore, Maryland, United States of America; 3 Department of Art as Applied to Medicine, Johns Hopkins School of Medicine, Baltimore, Maryland, United States of America; Ohio State University Medical Center, United States of America

## Abstract

**Background:**

Mitochondrial DNA (mtDNA) mutations are reported in different tumors. However, there is no information on the temporal development of the mtDNA mutations/content alteration and their extent in normal and abnormal mucosa continuously exposed to tobacco smoke in lung cancer patients.

**Methodology:**

We examined the pattern of mtDNA alteration (mtDNA mutation and content index) in 25 airway mucosal biopsies, corresponding tumors and normal lymph nodes obtained from three patients with primary lung cancers. In addition, we examined the pattern of mtDNA mutation in corresponding tumors and normal lymph nodes obtained from eight other patients with primary lung cancers. The entire 16.5 kb mitochondrial genome was sequenced on Affymetrix Mitochip v2.0 sequencing platform in every sample. To examine mtDNA content index, we performed real-time PCR analysis.

**Principal Findings:**

The airway mucosal biopsies obtained from three lung cancer patients were histopathologically negative but exhibited multiple clonal mtDNA mutations detectable in the corresponding tumors. One of the patients was operated twice for the removal of tumor from the right upper and left lower lobe respectively within a span of two years. Both of these tumors exhibited twenty identical mtDNA mutations. MtDNA content increased significantly (P<0.001) in the lung cancer and all the histologically negative mucosal biopsies except one compared to the control lymph node.

**Conclusions/Significance:**

Our results document the extent of massive clonal patches that develop in lifetime smokers and ultimately give rise to clinically significant cancers. These observations shed light on the extent of disease in the airway of smokers traceable through mtDNA mutation. MtDNA mutation could be a reliable tool for molecular assessment of respiratory epithelium exposed to continuous smoke as well as disease detection and monitoring. Functional analysis of the pathogenic mtDNA mutations may be useful to understand their role in lung tumorigenesis.

## Introduction

The human mitochondrial DNA (mtDNA) is a 16.5 kb double stranded closed circular molecule which codes for the 12S and 16S rRNAs, 22 tRNAs and 13 proteins essential for the mitochondrial respiratory complex [1]–[2]. Most human cells contain hundreds of copies of mitochondrial DNA (mtDNA) and nearly all of these mtDNA copies are identical i.e. homoplasmic at birth [1]–[2]. Mutation rate in mtDNA is approximately 10 times higher than nuclear genomic DNA (nDNA) [1]. To date, clonal mtDNA mutations have been reported in different tumors [3]. There is little information on the temporal development of these mutations and their extent in normal and abnormal mucosa exposed to tobacco smoke.

Lung cancer kills more than 1 million people worldwide with smoking being the most important risk factor [4]. In the United States, there were over 215,020 cases of lung cancer and an estimated death of 161,840 in 2008 [4]. Despite significant improvement in therapeutic modalities including surgery, platinum based chemotherapy and radiotherapy alone or in combination, the overall 5-year survival rate is only 15% [4]. Eighty-five percent of lung cancers occur in tobacco smokers [4]. Moreover, affected patients remain at significant risk for the development of second primary tumor throughout their lifetime. Thus, development of suitable methods for early disease detection, monitoring and continuous evaluation of the airways in the patients with primary lung cancer are of paramount importance.

In the present study, we examined the pattern of mtDNA alteration (mutation and DNA content) in airway mucosal biopsies obtained from follow up patients with primary lung cancers. Corresponding tumors and normal lymph nodes were also examined from these patients. All the biopsies appeared suspicious and abnormal under auto-fluorescence bronchoscopy, however they were histologically negative. But the map of the mtDNA alterations in these biopsies was striking and provided a unique insight into the extent of mitochondrial dysfunction and increasing risk of malignancy in patients who continue to smoke.

## Results

### Pattern of mtDNA mutation in the patients

**Figure 1:**
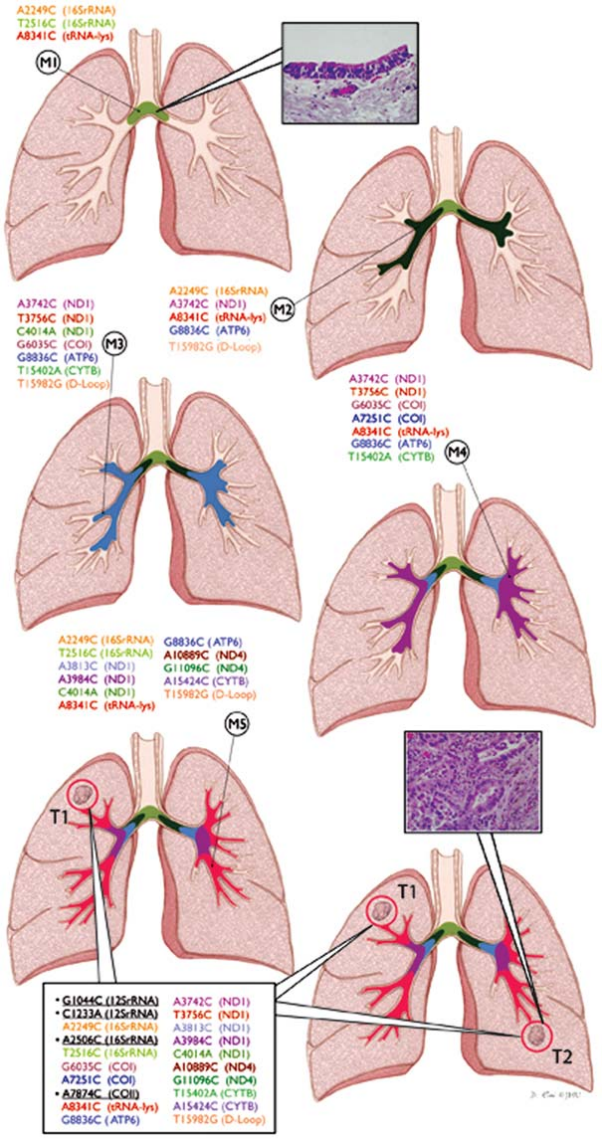
Pattern of mtDNA mutation in patient 1. The patient was operated twice on 2002 and 2004 with removal of tumors from the right upper (T1) and left lower lobe (T2) respectively. Five bronchoscopically abnormal airway mucosal biopsies were obtained following second surgery (T2) of this patient from main carina (M1), right upper lobe (M2 and M3) and left lower lobe (M3 and M4) surrounding both the tumors as depicted (Panel A–E). The mucosal biopsies exhibited a range of 3–11 mtDNA mutations as represented in different color (Panel A–E). Identical mutations are shown in the same color in different biopsies and the tumor. A different color code was also used for each mucosa to indicate the clonal progression of the lesions on both the lungs with accumulated mtDNA mutations. Representative histological photomicrograph of the mucosa and the tumor has been shown in panel A and F. Both the tumors T1 and T2 exhibited twenty identical mtDNA mutations (E–F, Bottom rectangle) including all the 16 mutations exhibited by the five mucosal biopsies (Matched color). The additional 4 mtDNA mutations detected in the tumors are represented underlined in black color in the bottom rectangle (Panel E–F). Two mtDNA mutations (*G8836C* and *A8341C*) were present in 4/5 and 3 other mutations (*A2249C*, *A3742C* and *T15982G*) were present in 3/5 mucosal biopsies. Tumors are encircled to indicate their development from the clonal mucosal patches. T1: Tumor from the right upper lobe; T2: Tumor from the left lower lobe; M: Mucosa.

**Figure 2:**
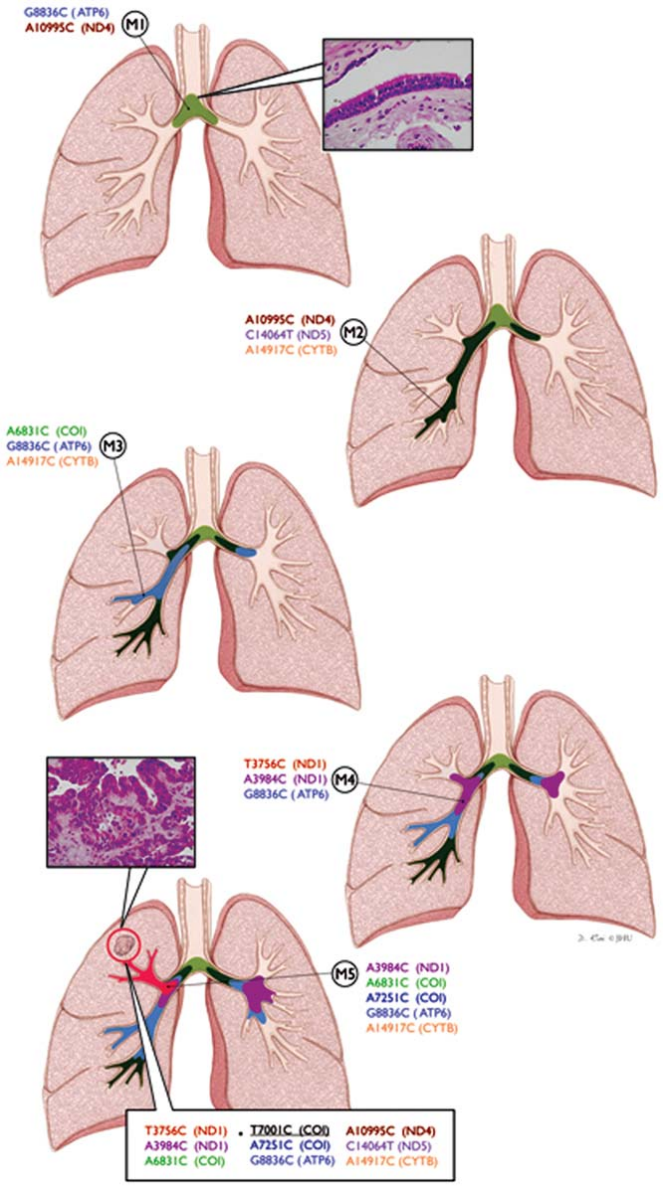
Pattern of mtDNA mutation in patient 2. The patient was operated on 2005 for surgical removal of tumor from the right upper lobe. Five bronchoscopically abnormal airway mucosal biopsies were obtained from main carina (M1), right lower lobe (M2 and M3) and right upper lobe (M3 and M4) surrounding the tumors as depicted (Panel A–E). The mucosal biopsies exhibited a range of 2–5 mtDNA mutations as represented in different color (Panel A–E). Identical mutations are shown in same color in different biopsies and the tumor. A different color code was also used for each mucosa to indicate the clonal progression of the lesions with accumulated mtDNA mutations. Representative histological photomicrograph of the tumor and normal mucosa are shown in Panel A and D. The tumor exhibited 9 mtDNA mutations (Represented in the Bottom rectangle) including all the 8 mutations exhibited by the five mucosal biopsies (Matched color). An additional mtDNA mutation (*T7001C*) detected in the tumor is underlined in black in the bottom rectangle. Tumor was encircled to indicate its development from the clonal mucosal patches. M: Mucosa.

Figure 1 shows the pattern of mtDNA mutations in patient 1. This patient was operated twice within a span of two years on both the lungs. The first tumor (T1) was surgically removed in 2002 from the right upper lobe followed by the removal of the second tumor (T2) in 2004 from the left lower lobe. Five airway mucosal biopsies were taken from the main carina (M1), right lower (M2 and M3) and left upper lobe (M4 and M5) surrounding the primary tumor sites on both the lungs as depicted. The mucosal biopsies were taken during the follow-up of this patient after surgical removal of the second tumor from the left lower lobe. All five mucosal biopsies were histopathologically without evidence of dysplasia, and yet exhibited a range of 3–11 mtDNA mutations (Fig. 1). A total of 16 mtDNA mutations were detected in the mucosa of this patient. The first biopsy from the main carina exhibited 3 mtDNA mutations (M1, Fig. 1A). The second biopsy exhibited 5 mutations including 2 overlapping mutations with M1 (*A2249C*, *A8341C*, matched color, Fig. 1A–B). The third biopsy exhibited 7 mtDNA mutations, including 3 overlapping mutations with M2 (*A3742C*, *G8836C* and *T15982G*, matched color, Fig. 1B–C). The fourth biopsy harbored 7 mtDNA mutations, including overlapping mutations with M3 (*A3742C*, *T3756C*, *G6035C*, *G8836C* and *T15402A*, matched color, Fig. 1A–D), M2 (*A3742C*, *A8341C* and *G8836C*, matched color, Figs. 2B and D) and M1 (*A8341C*, matched color, Fig. 1A–D). The biopsy taken adjacent to the tumor in the left lower lobe exhibited 11 mtDNA mutations including overlapping mutations with M1 (*A2249C*, *T2516C* and *A8341C*), M2 (*A2249C*, *A8341C*, *G8836C* and *T15982C*), M3 (*C4014A*, *G8836C* and *T15982G*) and M4 (*A8341C* and *G8836C*) biopsies (Fig. 1A–E, matched color). Both invasive tumors (T1 and T2) exhibited 20 identical mtDNA mutations including all the 16 mutations detected in the surrounding 5 normal mucosa (Fig. 1E–F) (Odds ratio 6.9×10^10^∶1). Thirteen of these 20 (65%) mutations were in coding regions (COI, COII, ATP6, ND1, ND4 and CYTB) and 7 (35%) occurred in non-coding regions (12SrRNA, 16SrRNA, tRNA-Lysine, D-loop) (Fig. 1, F, bottom rectangle). The additional 4 mutations detected in the tumors were represented in Fig. 1E (Bottom rectangle, black, underlined). Two (*G8836C* and *A8341C*) to three mtDNA mutations (*A2249C*, *A3742C* and *T15982G*) were present in 4/5 and 3/5 mucosa respectively and in both tumors (Fig. 1). Representative histological photomicrographs of the mucosa and tumor are shown in Figs. 1A and 1F. Different color codes correspond to a specific mutated mtDNA pattern showing the clonal spread of the mtDNA mutation throughout the mucosa and eventually given rise to the genetically related tumors (Encircled).

In patient 2, five mucosal biopsies were obtained from the main carina (M1), right lower lobe (M2 and M3) and right upper lobe (M3 and M4) surrounding the primary tumor on the right lung as shown in Figure 2. All the 5 lesions were histopathologically normal as seen in patient 1 but exhibited a range of 2–8 mtDNA mutations. The first lesion exhibited 2 mutations where as the second one exhibited 3 with one identical mutation shared with M1 (*A10995C*, Fig. 2A–B, matched color). The third lesion (M3) exhibited 3 mtDNA mutations with one overlapping mutation with M2 (*A14917C*) and M1 (*G8836C*) (Fig. 2A–C, matched color). In the forth lesion (M4), 3 mutations were detected with one overlapping mutation (*G8836C*) with M3 and M1 (Fig. 2A–D, matched color). The fifth mucosal lesion (M5) exhibited 5 mutations including overlapping mutations of M4 (*A3984C* and *G8836C*), M3 (*A6831C*, *G8836C* and *A14917C*), M2 (A14917C) and M1 (*G8836C*) (Fig. 2A–E, matched color). The primary tumor resected from the right upper lobe of this patient exhibited 9 mtDNA mutations including all the 8 mutations detected in the surrounding 5 apparently normal mucosa specimens. All of these mutations (9/9) were in the mitochondrial coding regions (ND1, COI, ATP6, ND4, ND5 and CYTB) of the mtDNA (Fig. 2E, bottom rectangle). The additional mutation (*T7001C*) detected in the tumor is shown in Figure 2E (Bottom right rectangle, black, underlined). One mtDNA mutation (*G8836C*) was present in 4/5 and another mutation (A14917C) was present in 3/5 mucosal tissue samples (Fig. 2). Both of these mutations were also detected in the corresponding tumor (Fig. 2E). Representative histological photomicrographs of the mucosa and the tumor are shown in Fig. 2A and E). Different color codes correspond to a specific mutated mtDNA pattern showing the clonal spread of the mtDNA mutation throughout the mucosa and eventually given rise to the genetically related tumor (Encircled). Four mtDNA mutations (*G8836C*, *T3756C*, *A3984C* and *A7251C*) were identical among patients 1 and 2 (Fig. 1–2).

Patient 3 was a non-smoker with a bronchoalveolar carcinoma and exhibited only 2 mtDNA mutations in the tumor (Table 1). No mutation was detected in the normal mucosa. One margin with metaplasia from this patient also did not show any mtDNA mutation (Table 1). These results suggest a fundamental difference in field cancerization and extent of clonal patches among a non-smoker tumor. We sequenced 8 other patients for mtDNA mutation and found a range of 1–9 mutations in the primary tumors compared to the control, most of which were from the coding regions of the mtDNA (Table 2). Other than somatic mtDNA mutations, we also detected few germ line mtDNA sequence variants in the tumors and corresponding mucosal biopsies of all the patients (Table 3).

**Table 1:** Pattern of mtDNA mutation in patient 3.

Nucleotide position	RCRS^1^	Normal (Lymph node)	Mucosa (Pooled)^2^	Negative for tumor^3^	Metaplasia	Tumor	Amino acid change	mtDNA region
15403	c	c	c	c	c	t	I-I	CYTB
15809	g	g	g	g	g	a	A-T	CYTB

1 Revised Cambridge Reference Sequence;

2 Mucosal biopsies from 5 different regions were pooled together because of small sample size;

3 Tumor adjacent histologically normal margin.

**Table 2:** Pattern of somatic mtDNA mutation in the 8 lung cancer patients.

Nucleotide Position	RCRS^1^	Normal^2^	Tumor	Amino acid change	mtDNA region
194	t	t	**c**	-	D-loop
920	t	t	**c**	-	D-Loop
5326	c	c	**a**	T-D	*ND2*
8494	t	t	**a**	A-D	*ATP8*
9115	t	t	**g**	L-V	*ATP6*
10871	t	t	**c**	P-S	*ND4*
13442	t	t	**g**	T-S	*ND5*
14831	a	a	**c**	A-P	*CYTB*
15551	g	g	**a**	L-E	*CYTB*

1 RCRS: Revised Cambridge Reference Sequence;

2 Normal Lymphocytes.

**Table 3:** The pattern of germ line mtDNA sequence variants in the lung cancer patients.

Nucleotide position	RCRS^1^	Normal^2^	Tumor	Mucosa	Amino acid change	mtDNA region	Remark
72	a	**g**	**g**	**g**	-	D-Loop	R^3^
2705	a	**g**	**g**	**g**	-	16SrRNA	N
7026	c	**t**	**t**	**t**	A-S	COI	R
8185	g	**c**	**c**	**c**	C-C	COII	N
11445	g	**a**	**a**	**a**	M-K	ND4	N
11465	a	**g**	**g**	**g**	L-V	ND4	R
15563	t	**c**	**c**	**c**	Y-H	CYTB	N

1 Revised Cambridge Reference Sequence;

2 Matched normal lymphocytes;

3 R: Reported; N: Novel as per Human Mitochondrial Database and relevant literature.

### Alteration of mtDNA content in the lung cancer patients

**Figure 3:**
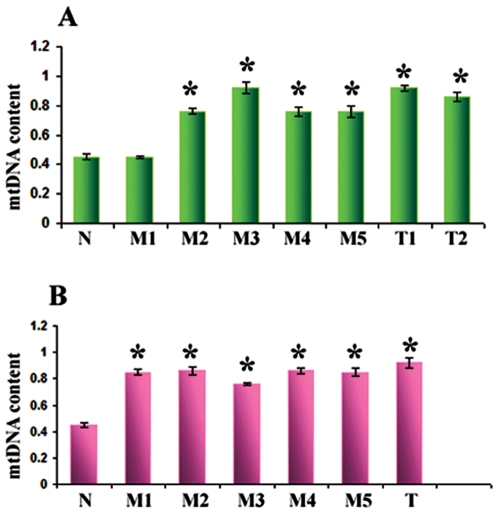
MtDNA content in the lung cancer patients. mtDNA content was measured by multiplex real-time PCR using nuclear encoded β-actin and mitochondria encoded COI gene. A ratio of COI/β -actin corresponds to the fold difference compared to control. MtDNA content increased significantly (P<0.001) in the mucosal biopsies and corresponding tumors compared to the normal control in both the patients as indicated. N: Normal lymph node; M: Mucosa; T: Tumor. *P value <0.05 compared to normal lymph node.

**Figure 4:**
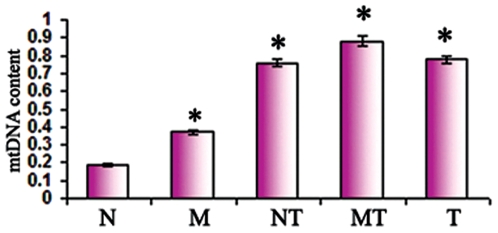
mtDNA content index in patient 3. mtDNA content increased significantly (P<0.05) in the mucosa (M), adjacent normal (NT) and in the metaplastic margin (MT) compared to normal lymph node used a s control.

We performed quantitative real-time PCR to determine mtDNA content in patients' mucosa, corresponding tumors, and non-neoplastic lymph nodes. With only a single exception in one biopsy (Patient 1, M1) mtDNA content was significantly higher (P<0.001) in the lung cancer and all of the histologically normal mucosal biopsies compared to the control lymph node (Fig. 3). This was even true in the mucosa and metaplastic margin identified in patient 3, the non-smoker (Fig. 4). MtDNA content was increased without a corresponding mtDNA mutation (Fig. 4). Thus, increased mtDNA content is a feature in extensive mucosal fields from both smoking and non-smoking lung cancer patients.

## Discussion

Mitochondrial DNA mutations are frequent in various tumor types [3], and detected in preneoplastic lesions thereby indicating their occurrence at early stages of multistage tumor progression [5]. Detection of mtDNA mutation is easier and more reliable compared to nuclear DNA because of their high copy number in cancer cells [6]–[7]. Therefore, scrutinizing mtDNA mutations in a small number of cells at different stages of tumor progression is possible. In order to use mtDNA mutation as an unbiased tool for detection of clonal cell populations, sequencing of the entire mitochondrial genome is necessary and must be done together with an appropriate non-neoplastic control because of the highly polymorphic nature of the mtDNA [7]. In the present study, amplification of considerable amount of mtDNA from very small mucosal biopsies, tumor and corresponding normal tissues enabled us to scan the whole mitochondrial genome. This was readily accomplished on the Affymetrix high throughput Mitochip v2.0 sequencing platform previously shown to be a reliable tool for detecting mtDNA mutation [5], [8]. This recent version of Mitochip (Compared to Mitochip v1.0) not only has a high sensitivity for mutation detection spanning the entire mitochondrial genome but also capable of determining heteroplasmic mtDNA mutation [8]–[9]. The observed mutation can also be verified by conventional sequencing. However, Mitochip v2.0 cannot detect small insertion/deletion as it is designed primarily to detect mtDNA mutation. The mutations detected in the present study were somatic or germ line in nature and confirmed as not due to any haplotypic variation [5], [8]–[9]. Thus, all the de novo mutations detected and reported in this study are associated with the tumor phenotype.

In patient 1, small follow up biopsies were taken from areas of the airway mucosa which appeared abnormal under Autofluorescence-Bronchoscopy. Although all the biopsies were histopathologically negative and without dysplastic changes, they exhibited multiple mtDNA mutations some of which were shared across large tracks of the airway epithelium. These results demonstrate the clonal spread of multiple mtDNA mutations throughout the respiratory mucosa. Clonal spread of p53 mutation and microsatellite alterations (LOH and MA) at multiple foci in normal appearing bronchial epithelium has been reported earlier [10]–[12]. In the present study, dramatic expansion of mutant bronchial epithelial clones was traceable through mtDNA mutation with great precision and in an unbiased manner by sequencing the entire mitochondrial genome in every sample.

**Figure 5:**
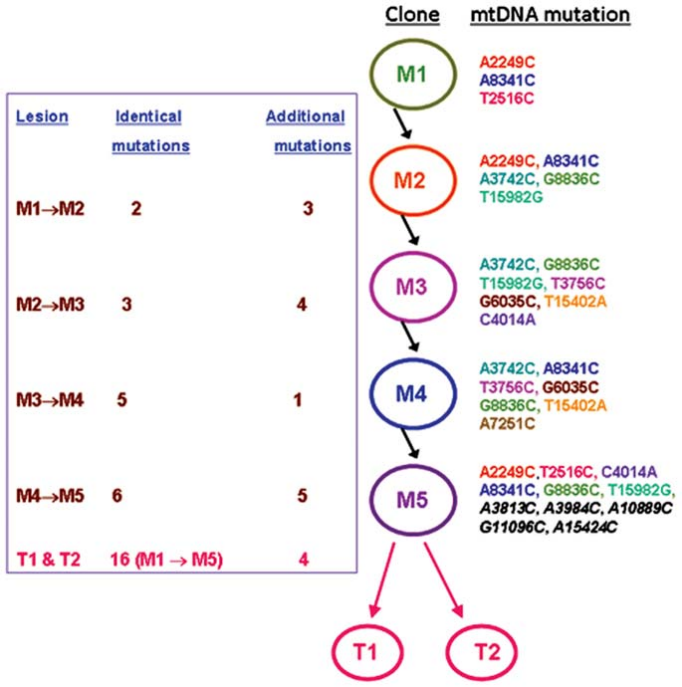
MtDNA genetic mutation tree in patient 1. Possible clonal evolution of the lung tumors has been depicted. Each mucosal biopsy (Circled, M1-M5) shared some identical mtDNA mutations (Matched color) along with additional mutations in the heterogeneous mucosal field. The fittest clones emerged in the primary tumors (T1-T2) with the more selective mtDNA mutations (Total 20 mtDNA mutations, 16 were shared between M1-M5 as in Figure 1).

It is likely that the biopsies contained heterogeneous patches of cells with clonal alterations acquired by random mitochondrial genetic drift [7]. As in patient 1, most of the clones shared a number of identical mtDNA mutations along with additional mutations necessary for the clonal expansion (Fig. 5). The fittest clones harboring more advantageous mtDNA/nDNA mutations progressed through the mucosa and eventually gave rise to two apparently independent primary tumors that are nevertheless surely linked by identical mtDNA mutations [7], [13]. Detection of all the mucosal mtDNA mutations in both the tumors resected within a span of 2 years strongly supports their clonal origin. Moreover, we have also shown that the two separate tumors from the opposite lungs exhibited multiple identical mtDNA mutations; the odds of this occurrence by chance are vanishingly low. The first tumor probably developed from one of the more dominant clones scattered on the right lung after acquiring the necessary mtDNA/nDNA alterations sufficient to promote tumorigenesis. The second tumor likely developed in the same way on the left lung but acquired nDNA mutational hits several months later or otherwise it could possibly be a pulmonary metastasis. The data from patient 2 also support the extensive clonal spread of mtDNA mutations and subsequent tumor development after acquiring sufficient mtDNA and/or nDNA changes. A high-resolution genome wide analysis of these biopsies and corresponding tumors revealed clonal alteration of a number of key nDNA encoded genes in these patients (Unpublished observation) further supporting this notion. However, due to unavailability, we could not examine the pattern of mtDNA alteration spectrum in a relatively large number of patients.

The presence of frequent germ line mtDNA sequence variants was suggested as an indicator of a high susceptibility genetic background which might facilitate concomitant somatic mutation in mtDNA and nDNA [14]. Detection of clonal germ line sequence variants and simultaneous somatic mtDNA mutations in tumors and mucosa of the lung cancer patients strongly support this notion.

Pathogenic mtDNA mutations may indicate for their functional contribution in progression of the tumors. Among the mtDNA mutations observed in these patients, the coding G8836C (ATP6) mutation is recently reported in patients with Leber Hereditary Optic Neuropathy (LHON)-like syndrome and thyroid tumors [15]–[16]. The interspecies conservation of this nucleotide is high and this mutation has been predicted to be pathogenic [15]. Notably, this mtDNA mutation was detected in 8/10 mucosal biopsies and all 3 tumors examined from both the patients supporting a functional contribution in tumorigenesis. However, further functional analysis is necessary to draw a more definitive conclusion. MtDNA mutation at identical nucleotide position among different lung and kidney cancer patients were reported earlier [16]. In the light of a pathogenic/contributing mtDNA mutation, a specific mtDNA mutation appeared to have been selected in different tumor types. We made similar observation in this present study and thus, occurrence of the pathogenic G8836C and other identical mtDNA mutations observed among different lung cancer patients are unlikely to be due to sample contamination. Notably, the G8836C pathogenic mtDNA mutation detected in LHON and thyroid cancer [15]–[16] and frequently in our study indicates for a functional role in lung cancer progression.

Most of these mutations were found in the coding regions of the mtDNA which could potentially lead to a disruption of the respiratory complex and result in an increase in Reactive Oxygen Species (ROS). Recent studies have shown that mtDNA mutations lead to an increase in ROS and promote tumor cell growth, proliferation and metastasis [17]–[20]. One study has shown an increase in mtDNA copy number in lung fibroblasts as an early event in response to oxidative stress [21]. Recently, an increase in mtDNA content was identified with aging and smoking associated lung-tissues and primary head and neck squamous cell carcinomas [22]–[23]. Histologically negative mucosal biopsies as well the tumors of both the patients exhibited significantly elevated level of mtDNA content. Thus, it appears that with the evolution of homoplasmic mtDNA mutation and clonal spread, mtDNA content also increases. Of note, some mutations (including *G8836C*) were identical in both the patients who were regular smokers, suggesting the effects of common targets from tobacco carcinogens on mtDNA. The high frequency of mtDNA mutations among smokers also suggests a key role for early mitochondrial genetic changes in the progression of their tumors.

In the present study, clonal progression of histologically normal appearing respiratory mucosa into tumor was traceable through mtDNA mutations. This is the most definitive study to demonstrate that the 2 tumors in the opposite locations in one patient (Patient 1) are clonally related. However, we could not examine more follow up patients due to the lack of bio-specimens. Documentation of these extensive clonal populations with mtDNA mutations sheds light on the challenges of early detection approaches. Moreover, targeting these mtDNA mutations may be helpful based on molecular detection in sputum and blood DNA in chemoprevention approaches and new biologic therapeutics. Further functional analysis of the common mtDNA mutations will allow us to better understand their specific role in cancer progression and chemoresistance.

## Materials and Methods

### Patients' history and lung specimens

Three follow-up lung cancer patients were examined for mtDNA mutation with signed informed consent in a Johns Hopkins IRB approved protocol. Patient 1 was a 75 year old female who underwent a right upper lobectomy in 2002 for a stage IIB poorly differentiated squamous cell carcinoma (T3N0MX). In 2004, she underwent a left lower lobectomy for a second stage IIB (T2N0MX) squamous cell carcinoma. Patient 2 was a 58 year old man, who in 2005 underwent a right upper lobectomy for a stage IA (T1N0MX) moderately differentiated squamous cell carcinoma. Both patients were regular smokers for more than 10 years. Patient 3 was a 48 years old female who underwent a right upper lobectomy for a stage IA (T1N0MX) bronchioloalveolar carcinoma. This patient was a non-smoker. During follow-up, airway mucosal biopsies were taken from areas suspicious for mild to moderate to severe dysplasia or carcinoma in situ (CIS) based on Auto-Fluorescence-Bronchoscopy (R.Y. and J.B.). All the biopsy specimens were taken at the same time. Biopsies were histologically evaluated by a lung pathologist (W.H.W). The surgically resected lung tumors were also obtained from each patient. As a control, matched normal lymph node free of tumor was used in each case. In addition, we also sequenced matched normal and tumor tissues from 8 other lung cancer patients (Table 4).

**Table 4:** History of the 8 lung cancer patients additionally sequenced for mtDNA mutation.

Patient ID	Age	Sex	Site^1^	Stage	Histology^2^	Tobacco^3^
21260	59	F	LLL	T2N0MX	SCC	NS
19312	75	F	RLL	T2N0MX	SCC	NS
11571	62	F	LUL	T1NOMX	SCC	60 PYH
655	78	F	RUL	T1NOMX	SCC	60 PYH
43620	70	M	RUL	T2N0MX	SCC	80 PYH
222	69	M	RUL	T2N0MX	SCC	100 PYH
15977	57	M	LUL	T2N1MX	SCC	40 PYH
14370	61	M	RLL	T1N0MX	SCC	100 PYH

1 RUL: Right upper lobe; RLL: Right lower lobe; LLL: Left lower lobe;

LUL: Left lower lobe; RML; Right middle lobe.

2 SCC: Squamous Cell Carcinoma;

3 All the tobacco positive patients have a smoking history of more than 10 years;

NS: Non Smoker; PYH: Pack year smoked.

### Mitochondrial whole genome amplification and sequencing

Genomic DNA was extracted according to our standard protocol from the microdissected tumor tissues and other specimens [5]. We amplified whole mitochondrial genomic DNA from 10 ng of genomic DNA template using REPLI-g mitochondrial DNA amplification kit according to the manufacturer's protocol as described earlier [8]. The amplified DNA was then purified using DNA MiniAmp Cleaning kit (Qiagen, Valencia). We examined the purity of the amplified mtDNA and nuclear DNA contamination by PCR analysis using primer specific for mitochondria encoded COXI/COXII and nuclear encoded β-actin. Four to five hundred nanogram of purified mtDNA was used for sequencing on the Affymetrix MitochipV2.0 platform [5], [8].

### Mitochip v2.0 sequencing array analysis

We performed fragmentation, labeling and Chip hybridization of the mtDNA as per Affymetrix protocol with appropriate controls as described earlier [5], [8]. Data analysis was performed using Affymetrix GSEQ software and the Revised Cambridge Reference Sequence (RCRS) was used as reference sequence [24]. The MitoAnalyzer software was also utilized to verify the mtDNA mutations at different nucleotide positions as described earlier [5], [8].Somatic mutations were identified as base pair changes in mtDNA when compared to normal mtDNA sequence found in lymph nodes free of tumor. Germ line mutations were identified as base pair changes present in the normal lymphocytes as well as tumor tissues and/or margin samples compared to the RCRS [14]. A germ line sequence variant was designated as novel when absent in the human mitochondrial database and other relevant studies in the same haplogroups [14], [25]. Sequence variants previously reported in different diseases including cancer are designated as pathogenic [14], [25].

### Quantitative real-time PCR

To examine mtDNA content, we used the 7900HT sequence detection system (Applied Biosystems, Foster City, CA) to amplify nuclear DNA (nDNA) encoded β-Actin and mtDNA encoded Cytochrome C oxidase I (COI) using genomic DNA template as described earlier [26].

### Statistical analysis

Student's *t*-test was performed to determine the statistical significance. P value less than 0.05 was considered significant. All P values generated were two sided.

### Odds ratio

The odds of 20 random identical mtDNA mutations occurring by chance in two separate tumors localized in the opposite lungs of the same patient were calculated as [(genome size)×(mutations)]×[(genome size)×(mutations)] i.e. [16,499∶1×20]×[16,499∶1×20] = 6.9×10^10^∶1 [27].
